# Supplementary material for: Socially driven negative feedback regulates activity and energy use in ant colonies
Source: PLoS Comput Biol. 2024 Nov 25;20(11):e1012623. doi: 10.1371/journal.pcbi.1012623 (PMC11649087; doi:10.1371/journal.pcbi.1012623)
Supplement: S1 File — (PDF) [file pcbi.1012623.s001.pdf]

Supporting file for "Socially driven negative feedback regulates activity and energy use in ant colonies" by Porfiri, Abaid, and Garnier

Text A. Adequacy of the scaling formulas. We compare equation set (4) with equation (2) for the parameter values listed in the Results sections "Model predictions for parameters calibrated on ant colonies" and "Monte Carlo simulations" ($N = 500$, $\alpha = 1.47$, $\gamma_0 = 1.21\,\mathrm{s}^{-1}$, and $\delta = 0.315\,\mathrm{s}^{-1}$), varying β_0 from $\gamma_0/10$ to $10\gamma_0$. For each value of β_0, we compute the relative error of the scaling formula with respect to the exact value (ϵ_A, ϵ_I, and ϵ_R, for $A^\star$, $I^\star$, and $R^\star$, respectively). Results in Fig Aa indicate that asymptotic predictions for any of the variables are within the same order of magnitude of the exact value for $\beta_0 \gtrsim 1$.

As a further test on the predictions of the scaling arguments, we vary N from 10 to 1,000 (typical size of an ant colony), while retaining the other parameter values as mentioned above. For each value of β_0, we minimize the squared error between the expressions in equation set (4) and allometries of the form $C_A N^{\rho_A}$, $C_I N^{\rho_I}$, and $C_R N^{\rho_R}$, where C_A, C_I, and C_R are proportionality constants for the power laws. Results in Fig Ab confirm the validity of the asymptotic expressions for $\beta_0 \gtrsim 1$, whereby scaling exponents are within approximately 10% of predictions in equation set (2) – theoretical predictions from equations (2)a,b would yield 0.77 for ρ_A and ρ_I and 1 for ρ_R.

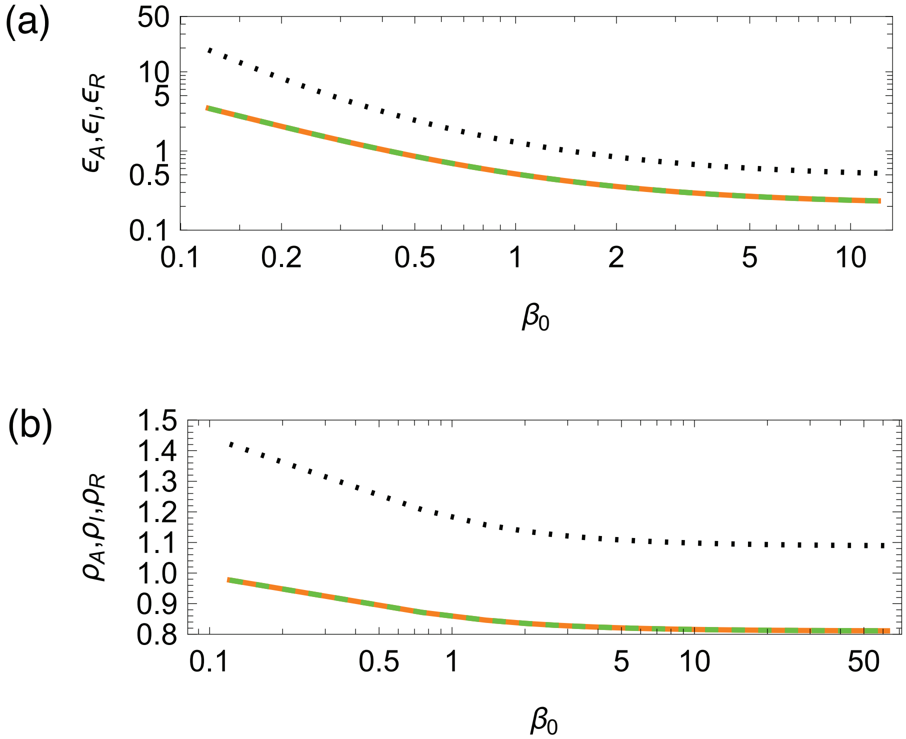

Figure A: Analysis of scaling formulas, with respect to predictions of steady-state values and allometries in the relationship between steady-state values and colony size, for different values of the social contagion parameter. Results are for model parameters for harvester ants. (a) Relative error in steady-state value for active (ϵ_A, orange solid), inactive (ϵ_I, green dashed), and refractory (ϵ_R, black dotted) ants for a colony of $N = 500$ individuals and model parameters. (b) Scaling exponents for active (ρ_A, orange solid), inactive (ρ_I, green dashed), and refractory (ρ_R, black dotted) ants obtained by fitting steady-state values using allometries for colony sizes ranging from $N = 10$ to 1000 and model parameters.
