# Supplementary material for: Socially driven negative feedback regulates activity and energy use in ant colonies
Source: PLoS Comput Biol. 2024 Nov 25;20(11):e1012623. doi: 10.1371/journal.pcbi.1012623 (PMC11649087; doi:10.1371/journal.pcbi.1012623)
Supplement: Attachment — Submitted filename: Epidemic ants PLOS Comp Bio response.pdf [file pcbi.1012623.s002.pdf]

NYU | TANDON SCHOOL OF ENGINEERING

M. Porfiri, Ph.D.

Comments from the Editor

This is an interesting and well-motivated paper that examines the potential role of negative feedback signals in regulating activity levels in foraging social insect colonies, leading to a metabolic scaling-like law for increasing colony size. Previous work on negative feedback in foraging social insect colonies has proposed its role in signalling danger (Nieh and colleagues), lack of profitability (Robinson and colleagues), aiding decision dynamics (Seeley and colleagues), and suppressing variation in performance (Reina and Marshall). The present manuscript thus makes an interesting complementary contribution, albeit one slightly foreshadowed in the literature, as discussed below).

1. While the presentation is generally clear, reviewer 1 feels that the key scaling law argument could be presented graphically. While I feel that the authors' present figure 3 does this to a degree, I agree with the reviewer that it could be presented in other, more intuitive ways. I would also suggest that some of the detail of the derivations could be moved into supplementary material, since the primary audience will be biologists, and studies show that equations inversely correlate with citation rates in the life sciences (Fawcett and Higginson, PNAS 109 (29) 11735-11739).

   Response: *Thank you for this perspective on how to make our work more accessible to diverse audiences. We added a new figure (Fig. 2 in the revised manuscript) to graphically demonstrate our main result about the scaling exponent, with a more detailed explanation in the caption.*

2. In addition, I have some comments of my own on the relationship between the present manuscript and existing work in the field - the authors note the existence of the above prior work on negative signalling, but in proposing their new model I am not sure they fully appreciate its relationship to previous models, in which energetic considerations of negative feedback have explicitly been alluded to. The model of the present manuscript is essentially equivalent to the honeybee stop-signalling model of Seeley et al. with two changes - one is the collapse of a decision problem into a single nest site problem, the other is the introduction of an intermediate 'recovering' class for recipients of negative signalling, bringing the model closer to the epidemiological literature it has long paralleled. …This would help highlight the relationship with previous work where, in the binary decision case of the honeybee model, in another paper the authors explicitly discussed the energetic impact observed for negative feedback (first paragraph of Discussion of Pais et al., cited), where they also discuss a possible trade-off. While the present study makes standalone contributions, these links to the previous literature would strengthen it, in my opinion. I also felt that other work on regulation of energetic expenditure by social insect

colonies in the absence of negative feedback, for example the work of Deborah Gordon, could be cited.

Response: *We have added additional references in the introduction with comments on the role of negative feedback in regulating various forms of collective behavior in social insects.*

3. From pen and paper maths I didn't quickly convince myself that the introduction of the recovering class would qualitatively change the authors' conclusions on energetic scaling, and it might be interesting to also analyse the simpler model to see if this is the case.

   Response: *The refractory class in our model is critically important to recover the hypometric scaling of the number of active ants with colony size that we have observed in experimental data. When only two states (that is, active and inactive) are allowed, the number of ants in each class scales with N in the equilibrium state. This is in stark contrast to our main result in Eq. (5), which shows hypometric scaling of the number of active ants with N, consistently with the experimental results. We have included a new section in the Materials and Methods of the revised manuscript with the equations of this model and its equilibria to make this point ("Compartmental model without refractory state"), and add a comment to the Results ("Scaling") to highlight this point.*

4. Finally, it may interest the authors to note that they could substantially automate aspects of the derivation and analysis of systems such as their present model, as well as disseminate effectively, through the use of an open source tool (mum.readthedocs.io) - a brief notebook analysing their model is available from the Editor on request.

   Response*: Thank you for this information. We have shared our code, which is written in Mathematica, on a github repository https://github.com/dynamicalsystemslaboratory/Ant-Epidemic-Model, with this link shared in the revised manuscript. Readers will be able to freely read our code using Wolfram's free MathReader software regardless of purchasing a Mathematica license.*

Comments from Reviewer 1

The paper shows that modelling ant foraging dynamics with a simple model of information spreading that combines positive and negative feedback can give interesting results on system size scaling. The author's model shows that having nonlinear (social) negative feedback leads to different dynamics than only having linear (nonsocial) negative feedback. They also discuss that it can be interesting to look at this scaling in terms of the colony's energetic consumption. I agree with the authors that this problem is interesting, worth studying, and relatively understudied.

1. I imagine (from what I could understand) that the key point of the article is the hypometrical growth of active-vs-total group size. This is interesting and worth discussing, so I am in favour of the eventual publication of this article, however, I would like first to see the hypometrical growth results presented more clearly. Now this result is only (possibly) hidden in fig. 3 with the orange line slope lower than 1, or by eyeballing 5(a) and 5(b).

   My suggestion (but authors can come up with better solutions too) is to show two figures: (1) easy interpretation of comparing a set of scaling lines of A (vs N on x-axis) for selected relevant parameters
   (2) full space exploration, e.g., through a heatmap of the scaling slope of A (i.e. a heatmap of the slopes of the orange lines of fig.3) for all the range of parameters changed on the two axes (possibly beta and alpha).

   Response: *Thank you for this idea, which has prompted us to create a new figure (Fig. 2 in the revised manuscript), to demonstrate how the scaling exponent depends on the network connectivity.*

2. Authors indicate (l. 116) that "we neglect spontaneous activation and inactivation I -> A and A -> R" in order to "reduce the number of model parameters and focus on a minimalistic model". Previous analyses of similar models of opinion dynamics have shown the importance of spontaneous (i.e. nonsocial) state change (e.g. noisy voter models, Herrerías-Azcue & Galla Phys. Rev. E 2019, Reina et al. Comm.Phys. 2023), leading to qualitative changes of the mean-field dynamics. Could it also be the case with this model?

   Response: *Thank you for the suggestion, which indeed captures some of the lines of work we are currently pursuing. Unfortunately, reproducing our full analytical results for the model with spontaneous activation and deactivation is presently not feasible but all numerical evidence we gathered so far are supportive of our claims even when adding these processes.*

   To demonstrate this, we plot in Fig. L1 the numerical predictions from the compartmental model in equation set (1) with the addition of spontaneous activation (a term 0.1I(t) added to equation 1a and subtracted from equation 1b) and deactivation (a term 0.1A(t) added to equation 1c and subtracted from equation 1a) – note that the selected weights for these spontaneous terms on the same order as social and reverse social contagion to fairly weight these different drivers of the dynamics. In all cases, the behavior of the model is

qualitatively identical to the model simulated in the manuscript where spontaneous activation and deactivation are not considered, with the only appreciable difference being in the steady state values of the top case where reverse social contagion is dominant.

Similarly, we can vary the value of N and numerically compute the steady state values of A, I, and R, shown in Fig. L2, with $\beta_0/\gamma_0 = 10$ to facilitate comparison with Fig. 4 in the revised manuscript (formerly Fig. 5). We can see that the scaling exponents have the same pattern as in the case without spontaneous activation and deactivation, in that R scales approximately isometrically with N while A and I scale hypometrically.

Due to the purely numerical nature of these observations and our intention to continue working on the model expansion to include these phenomena, along with others like a fixed refractory period, we would prefer to not include these analyses in the present manuscript.

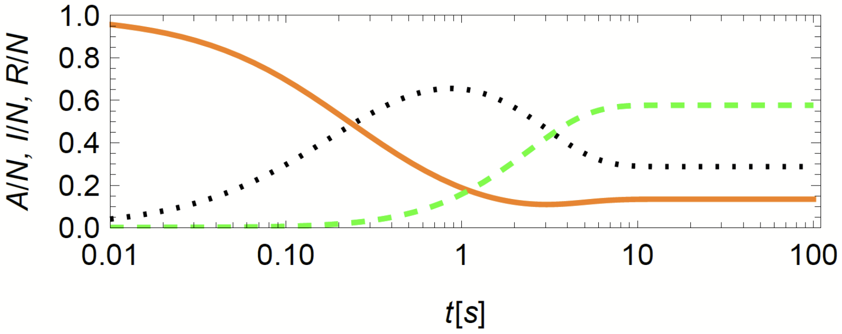

(a)

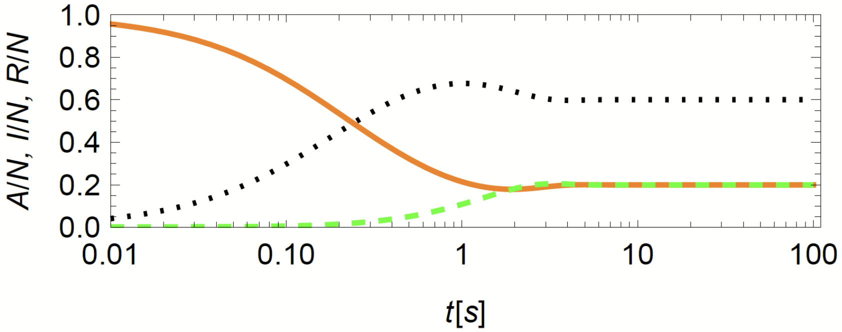

(b)

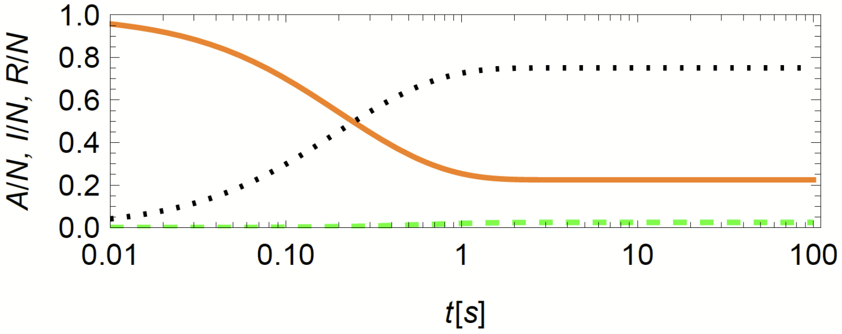

(c)

Fig L1. Numerical predictions from compartmental model in equation set (1) of the main manuscript with spontaneous activation and deactivation. Curves show the time-evolution of the fraction of active (*A*, orange solid), inactive (*I*, green dashed), and refractory (*R*, black dotted) ants for a colony of *N*=500 individuals and model parameters inspired by harvester ants plotted with time on a logarithmic scale. (a) Reverse social contagion dominating social contagion (β_0/γ_0 =1/10). (b) Reverse social contagion and social contagion at the same level (β_0/γ_0 =1). (c) Social contagion dominating reverse social contagion (β_0/γ_0 =10).

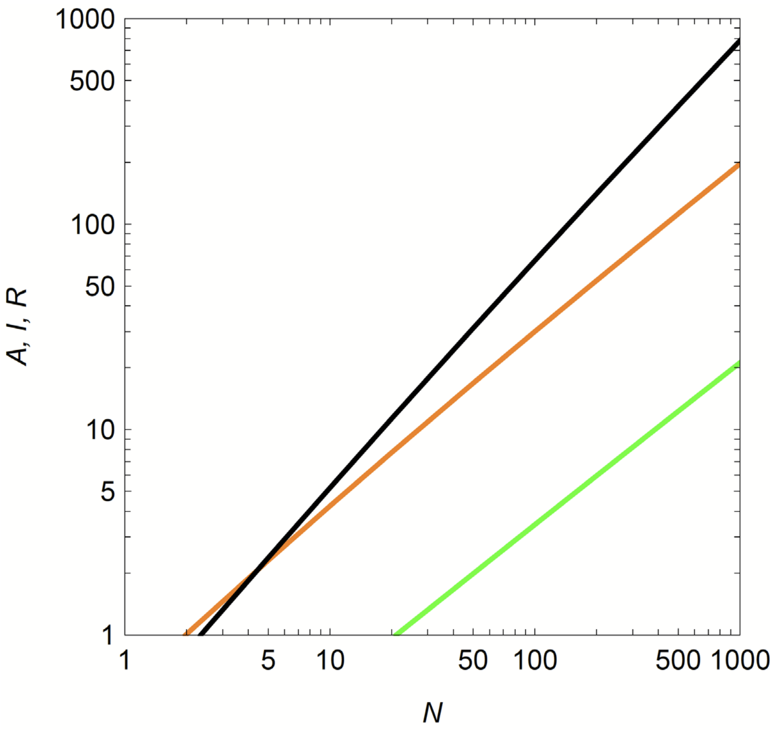

Fig L2. Numerical predictions for the steady-state number of active (*A*, orange), inactive (*I*, green), and refractory (*R*, black) ants as a function of the colony size, *N* , for model parameters inspired by harvester ants and β_0/γ_0 = 10.

3. The authors say (in the discussion) "inactivation mechanisms proposed in existing models of activity dynamics. Indeed, the latter are typically not socially driven". Please also comment on models that included socially-driven inactivation systems, e.g. [26, 29], and other stop signalling models (e.g. Bidari et al. RS Open Science 2019) or negative pheromone studies (e.g. Elva Robinson's work).

   Response: *We have added additional references in the introduction with comments on the role of negative feedback in regulating various forms of collective behavior in social insects.*

4. Simulation method. The authors use an Erdos Renyi network that they rewire each timestep, given that the topology is randomly changed at each timestep, what is the difference between using an ER net and the more widely spread approach of sampling one random neighbor from the full network? (i.e., random pairwise interaction, with state change triggered with state transition probabilities). If there is no reason for doing so, it looks like an unnecessary complication of the model which should be removed (as model simplicity is stated as one of the contributions). Otherwise, if there is a reason (e.g. ER allows you to study the impact of and to appreciate different scaling on k with different levels of hypometric growth); then, this result should be shown as it's very interesting.

Response: *In the current analysis, we draw a random network at each time-step* **Δ***t in which the probability of having a link between two nodes is p=<k>/N so that the average degree is always <k>. An alternative to this approach would be the pairwise one you are suggesting, in which one would consider a smaller time-step* **Δ***t/N and at each of these smaller steps, they would draw two nodes at random and connect with probability p N (which must be less than one by definition). For* **Δ***t and p sufficiently small, your proposal and ours would yield equivalent results – matching our theoretical predictions – as seen below. Compared to the pairwise update, we believe that our protocol is: i) more realistic as in reality all ants can respond to others at all times, and ii) more mathematically grounded as the time step and the average degree are not required to be small. As you can see in Fig. L3, the results are analogous for a small network but if you consider a large one, the pairwise update will not work as one cannot choose a value of p less than one that would match the average degree (in our example of N=500, <k>~4). A potential way around the issue of small time step could be to update pairs one by one, asynchronously, but this would not address the other issue of dealing with networks that may have large average degrees.*

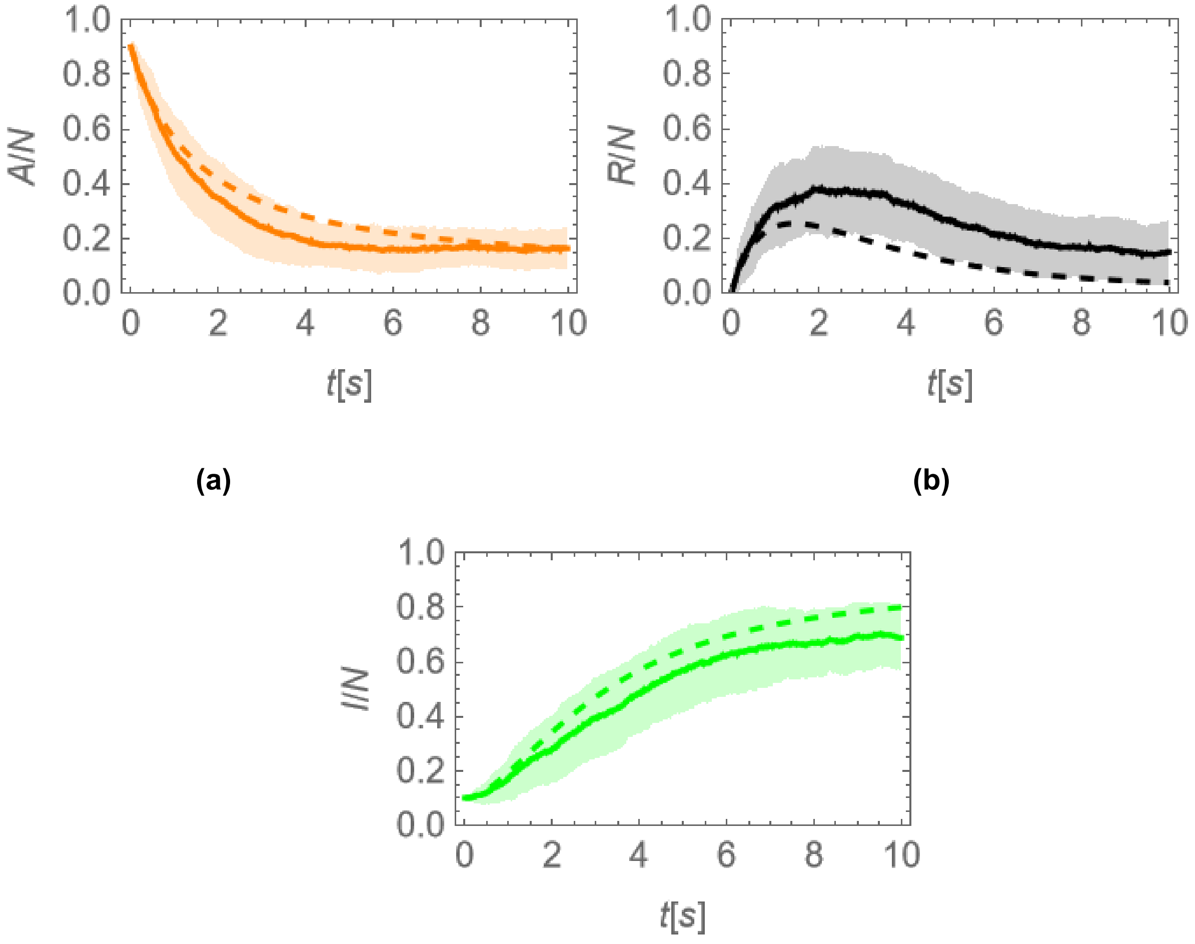

(c)

Fig L3. Comparison between the numerical results of the compartmental mode (dashed line) and simulations of the “asynchronous” agent-based model (solid line, mean, and dashed regions, one standard deviation above and below the mean) for a colony of N = 10 individuals. (a) Fraction of active ants (A, orange). (b) Fraction of inactive ants (I, green). (c) Fraction of refractory ants (R, black).

5. Results issues: l. 227 - “with initial conditions A0 = N − 1, I0 = 1, and R0 = 0 in fig.2, at t=0, R0 is higher than I0. This is confusing, please revise.

   Response: *Thanks for catching this source of confusion. Since a logarithmic scale was more appropriate for the horizontal axis of these plots to demonstrate the convergence to equilibrium, we plotted over a time range that did not include t=0. If the initial time and plot range are much reduced, we can see that the plots for I and R do indeed cross shortly after t=0. We agree with the reviewer that this could be confusing and added text to the caption of Figure 2 to clarify this point.*

6. l. 424 (Text S1) - unknown what C_A C_I and C_R are. Please explain.

   Response: *These terms are coefficients for the proportionality between powers of N and the variables A, I, and R. Since this work is focused on the scaling relationship rather than the exact expressions for these relationships, we neglected to define these terms. We added definitions in the revised version of the manuscript for clarity.*

7. Presentation issues: fig 1a is identical to fig.1 of the previous PNAS Nexus article.

   Response: *The figure is indeed reprinted from our earlier article, using the correct citation for the journal’s requirement and the paper copyright. Since this work is based on the same phenomenon of reverse social contagion, we opted to reprint this figure rather than recreate it.*

8. The section "Compartmental models" can be reduced in length as the mathematical steps for ODE stability analysis should go in the methods sections and the results text could focus more on presenting the model and discussing stability results, eq. (3) and (5), rather than deriving them.

   Response: *We appreciate this suggestion and have moved details on the derivation of the equilibrium to the Materials and Methods.*

9. l. 277 “top and bottom rows” -> “left and right columns”?

   Response*: You are correct. Thank you for catching this mistake which was held over from a previous version of the figure layout.*

Comments from Reviewer 2

The authors argue and demonstrate using results from a previous study on effect of reserve social contagion and stability analysis of a set of mathematical equations that the energy spending in ant colonies grows proportionally slower than the group size. The study offers a potential answer

to how large social groups regulate their activities (or energy spending in this case) without a centralized control system and makes a valuable contribution in this field.

Response: *Thank you for the concise and accurate summary of our work.*
